# Supplementary material for: A hole-selective hybrid TiO2 layer for stable and low-cost photoanodes in solar water oxidation
Source: Nat Commun. 2024 Nov 1;15:9439. doi: 10.1038/s41467-024-53754-9 (PMC11530438; doi:10.1038/s41467-024-53754-9)
Supplement: Supplementary file 1 — Supplementary Information [file 41467_2024_53754_MOESM1_ESM.pdf]

Supplementary Information

A Hole-Selective Hybrid TiO_2 Layer for Stable and Low-Cost Photoanodes in Solar Water Oxidation

Sanghyun Bae,[1,2] Thomas Moehl,[1] Erin Service,[1] Minjung Kim,[2] Pardis Adams,[1] Zhenbin Wang,[1] Yuri Choi,[2] Jungki Ryu[2,3] and S. David Tilley[1*]*

[1]Department of Chemistry, University of Zurich, Winterthurerstrasse 190, 8057 Zurich, Switzerland.

[2]School of Energy and Chemical Engineering, Ulsan National Institute of Science and Technology (UNIST), Ulsan 44919, Republic of Korea

[3]Center for Renewable Carbon, Ulsan National Institute of Science and Technology (UNIST), Ulsan 44919, Republic of Korea

*To whom correspondence should be addressed: david.tilley@chem.uzh.ch (S.D.T.); jryu@unist.ac.kr (J.R.)

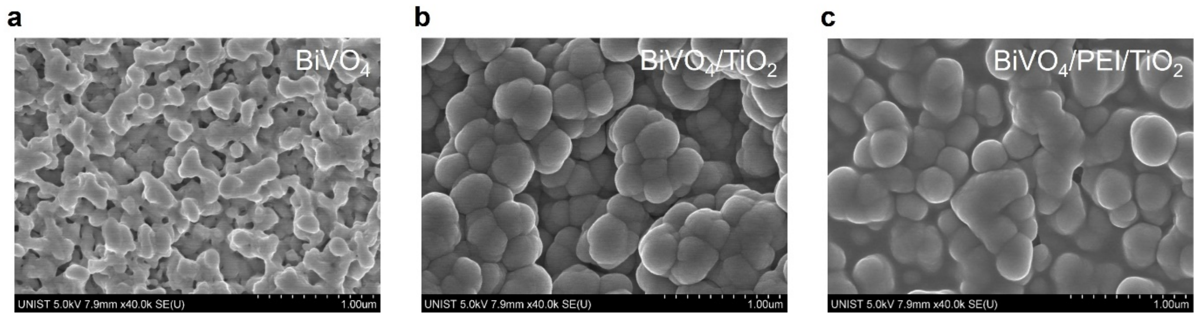

Supplementary Fig. 1. **Morphological analysis of photoelectrodes.** SEM image of $BiVO_4$ (**a**), $BiVO_4/TiO_2$ (**b**), and $BiVO_4$/PEI/TiO_2 (**c**). The thickness of the TiO_2 layer is 100 nm.

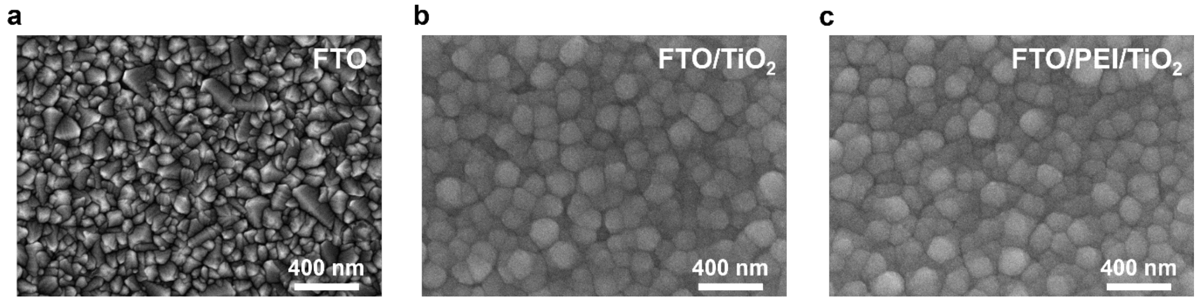

Supplementary Fig. 2. **Morphological analysis of FTO electrodes.** SEM images of FTO (**a**), FTO/TiO_2 (**b**), and FTO/PEI/TiO_2 (**c**). FTO/TiO_2 and FTO/PEI/TiO_2 were prepared in the same manner as the $BiVO_4$ photoanode. The PEI layer was deposited on the FTO substrate using spin-coating, followed by the deposition of 100 nm a-TiO_2 on the substrate.

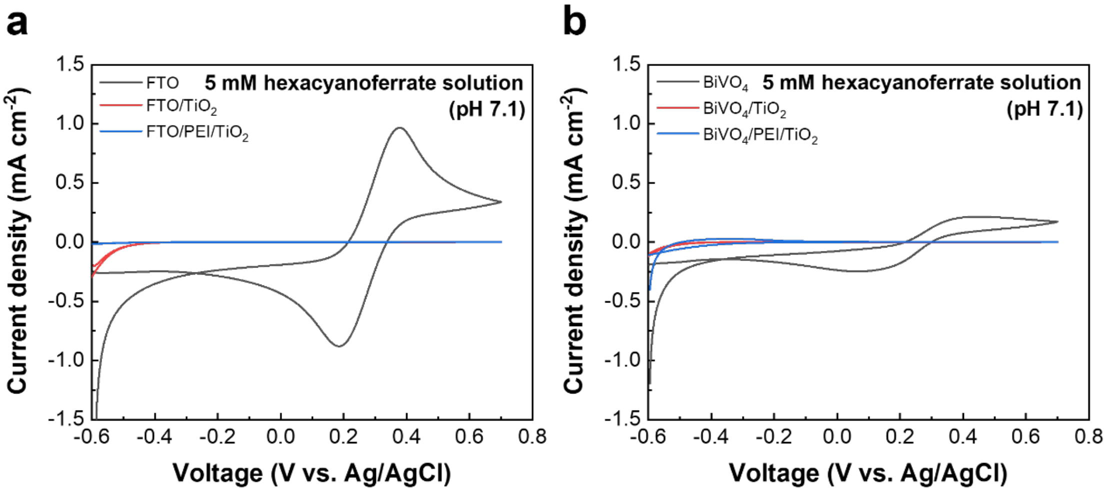

Supplementary Fig. 3. **Pinhole test using a ferricyanide solution.** Pinhole test was carried out using the cyclic voltammetry (scan rate: 50 mV s^{-1}) in the ferricyanide solution including 5 mM $K_4[Fe(CN)_6]$ and 5 mM $K_3[Fe(CN)_6]$ dissolved in 0.5 M KCl solution (pH 7.1). In the test, neither the TiO_2 nor the PEI/TiO_2 overlayers exhibited a redox peak for ferricyanide, which indicates that the FTO (**a**) and the $BiVO_4$ (**b**) surfaces were fully covered by the overlayers.

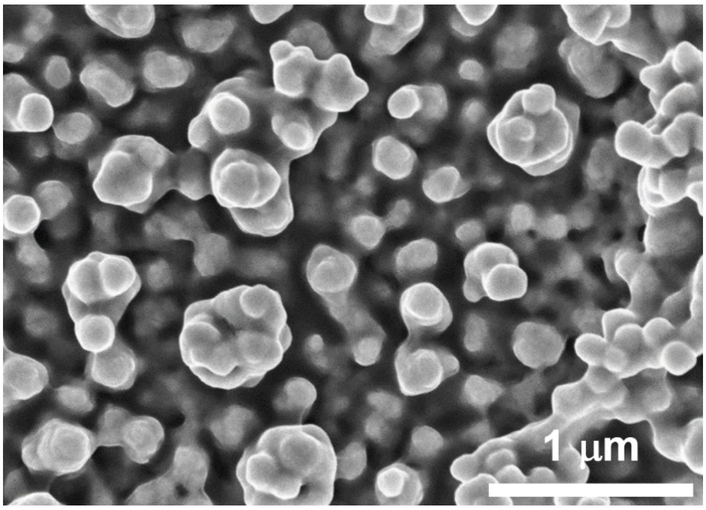

Supplementary Fig. 4. **Morphological analysis of $BiVO_4$/PEI photoanode.** SEM image of $BiVO_4$/PEI. The PEI was deposited on $BiVO_4$ by spin-coating method with 4 wt.% PEI solution.

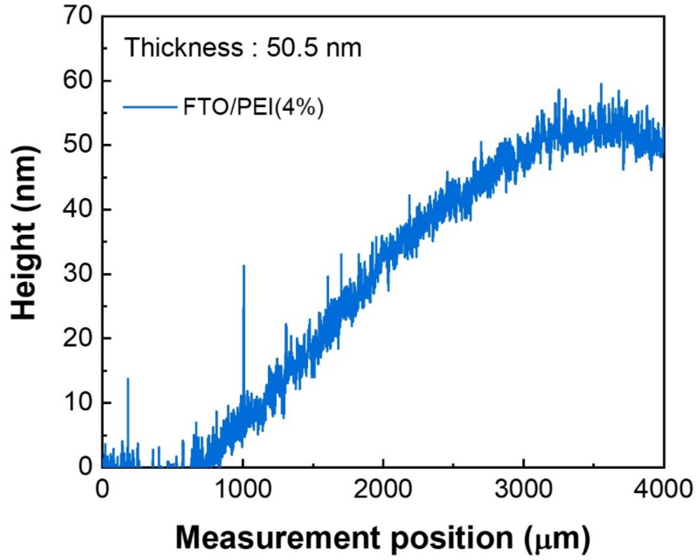

Supplementary Fig. 5. **Surface profile measurement.** Surface profilometry for measuring a thickness of the PEI layer on FTO substrate. The PEI layer was prepared by spin-coating using same concentration of PEI solution for $BiVO_4$ substrate.

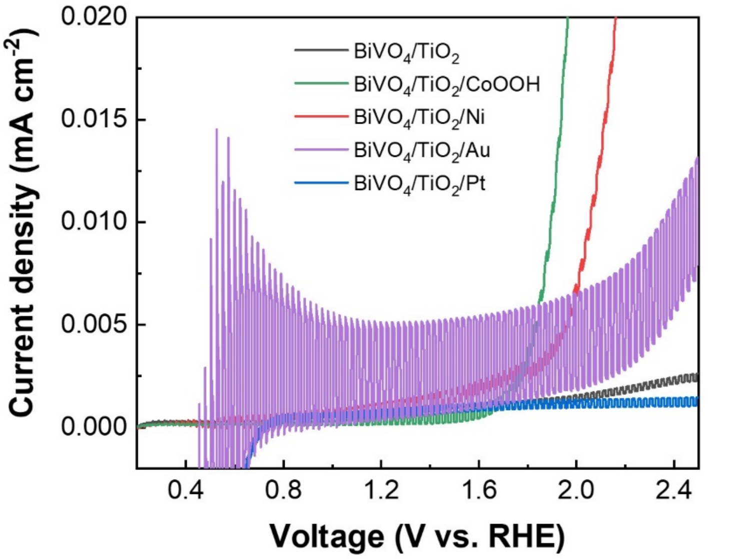

Supplementary Fig. 6. **Photocurrent measurement of $BiVO_4/TiO_2$ with and without co-catalysts.** PEC performance of $BiVO_4/TiO_2$ photoanode modified with various metal co-catalysts. The work function of each metal is as in the following: Ni (5.22 eV), Au (5.47 eV), and Pt (5.64 eV).

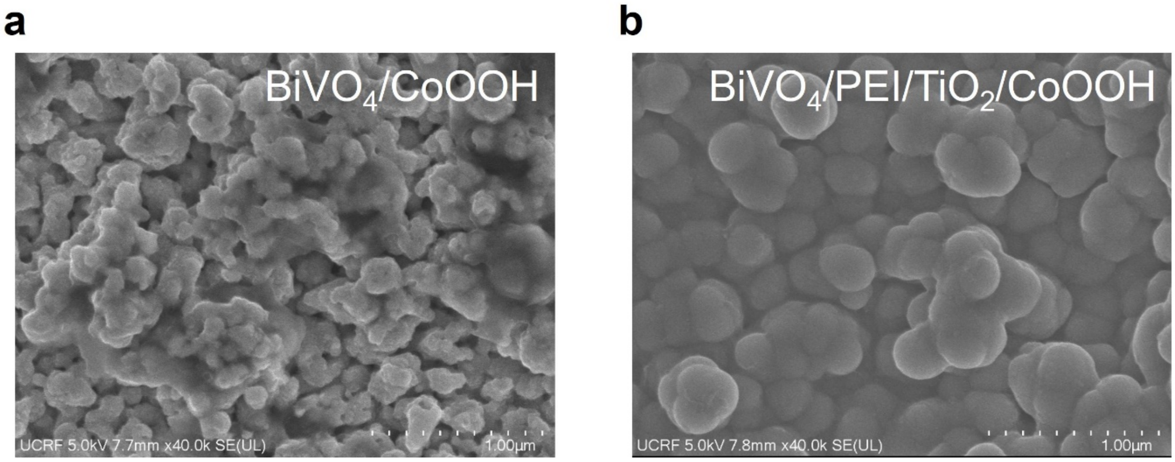

Supplementary Fig. 7. **Morphological analysis of photoanode with CoOOH.** SEM image of $BiVO_4$/CoOOH (**a**) and $BiVO_4$/PEI/TiO_2/CoOOH (**b**).

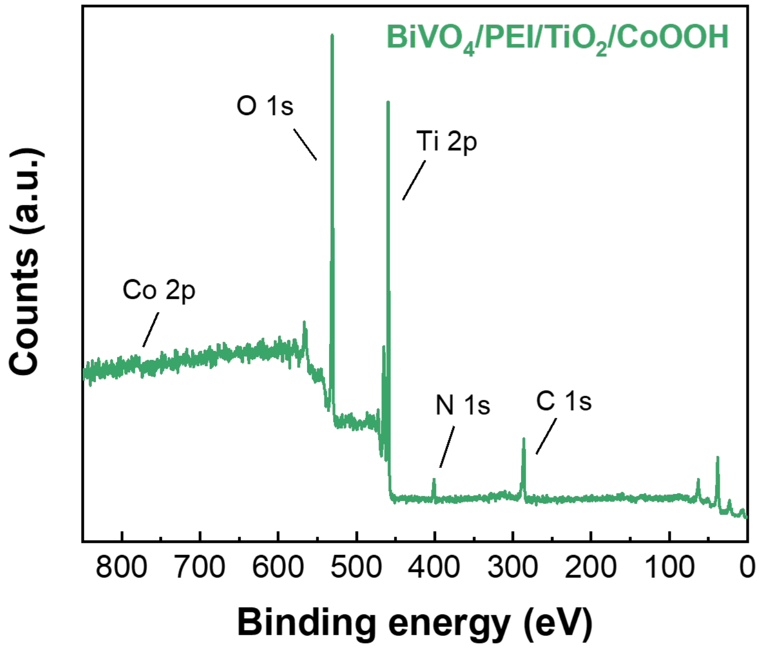

Supplementary Fig. 8. **XPS analysis of $BiVO_4$/PEI/TiO_2/CoOOH.** High resolution XPS spectra of $BiVO_4$/PEI/TiO_2/CoOOH. XPS analysis showed a Co 2*p* peak around 782.2 eV.

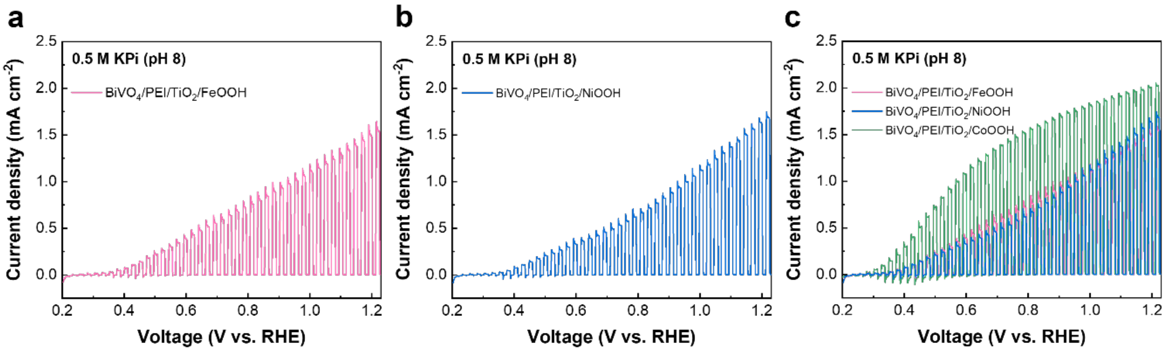

Supplementary Fig. 9. PEC water oxidation performance of $BiVO_4$/PEI/TiO_2 with various co-catalysts. LSV curves of $BiVO_4$/PEI/TiO_2 with FeOOH (**a**) and NiOOH (**b**) co-catalysts. $BiVO_4$/PEI/TiO_2/CoOOH (**c**) exhibited superior PEC performance in terms of onset potential and photocurrent compared to the other co-catalysts.

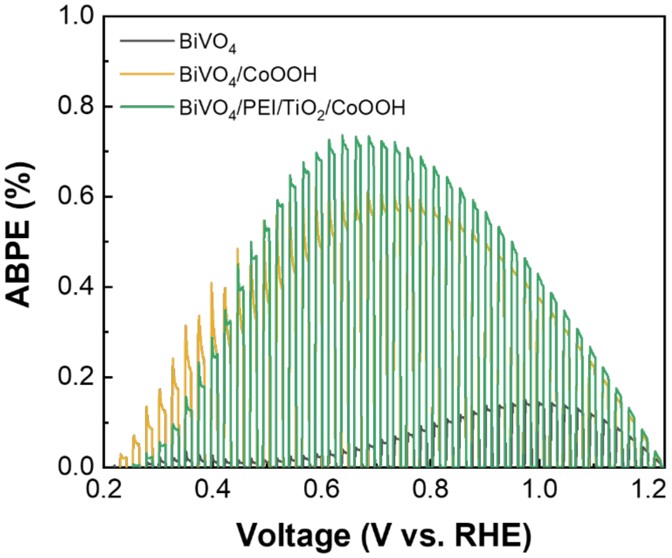

Supplementary Fig. 10. ABPE of $BiVO_4$, $BiVO_4$/CoOOH, and $BiVO_4$/PEI/TiO_2/CoOOH. The half-cell ABPE values was converted from photocurrent densities of each photoanode in the LSV curve to determine thermodynamically based conversion efficiency at a given applied potential.

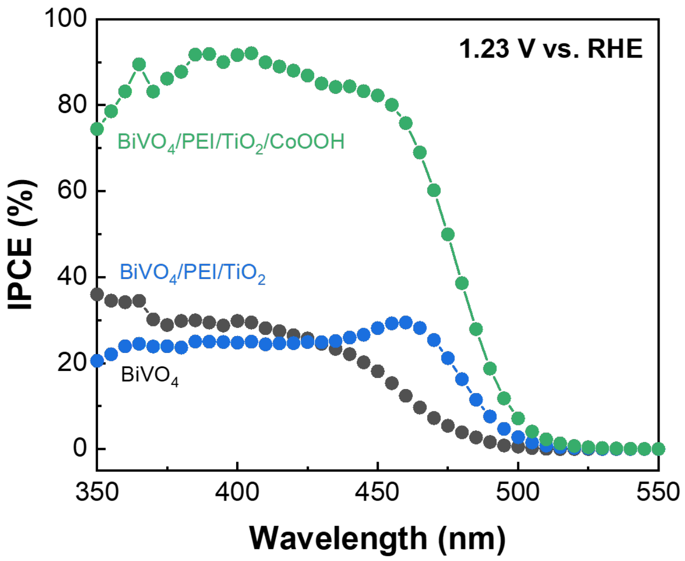

Supplementary Fig. 11. **IPCE of $BiVO_4$, $BiVO_4$/PEI/TiO_2, and $BiVO_4$/PEI/TiO_2/CoOOH.** The IPCE measurement was carried out in 0.5 M KPi (pH 8) at 1.23 V vs. RHE under 10% white light illumination. The integrated photocurrent density of $BiVO_4$ (1.02 mA cm^{-2}) matched well with that from the LSV curve, while $BiVO_4$/PEI/TiO_2 (1.33 mA cm^{-2}) and $BiVO_4$/PEI/TiO_2/CoOOH (4.08 mA cm^{-2}) exhibited higher integrated photocurrent densities compared to their LSV measurements, which indicates a limitation at higher light intensities.

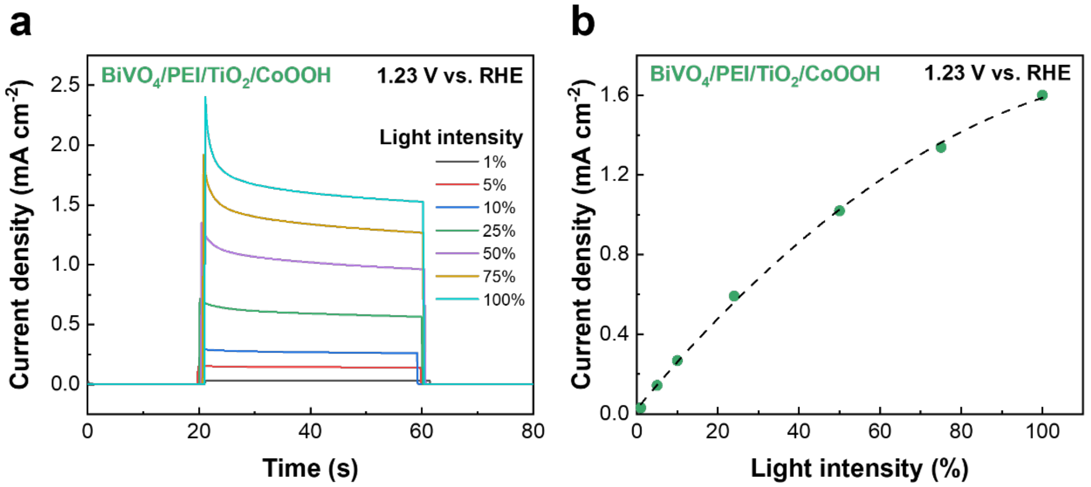

Supplementary Fig. 12. Light intensity dependence of the photocurrent in $BiVO_4$/PEI/TiO_2/CoOOH. Chronoamperometry (CA) of $BiVO_4$/PEI/TiO_2/CoOOH (**a**) was carried out in 0.5 M KPi (pH 8) under white light (1% ~ 100% of 1 sun illumination by white light LED). The photoanode showed a decreasing slope of photocurrent improvement with increasing light intensity (**b**), indicating limitations of the catalytic activity of CoOOH at higher light intensities.

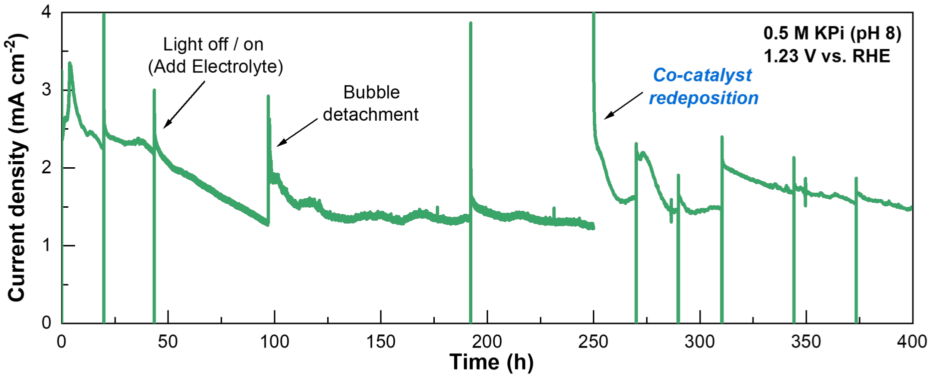

Supplementary Fig. 13. Long-term stability test of $BiVO_4$/PEI/TiO_2/CoOOH. CA measurement of $BiVO_4$/PEI/TiO_2/CoOOH in 0.5 M KPi electrolyte (pH 8) at 1.23 V vs. RHE. After 250 h stability test, the cobalt co-catalyst was redeposited, followed by an additional 150 h stability test.

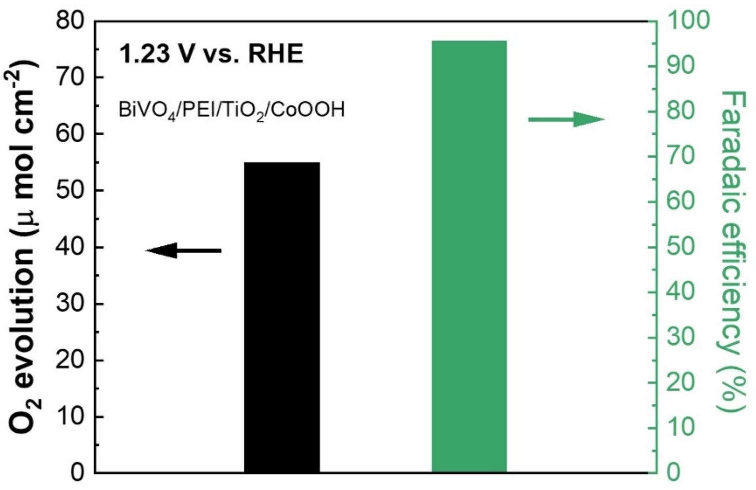

Supplementary Fig. 14. Faradaic efficiency of $BiVO_4$/PEI/TiO_2/CoOOH. GC measurement of $BiVO_4$/PEI/TiO_2/CoOOH photoanode. The measurement of oxygen evolution was carried out after a 3 h stability test.

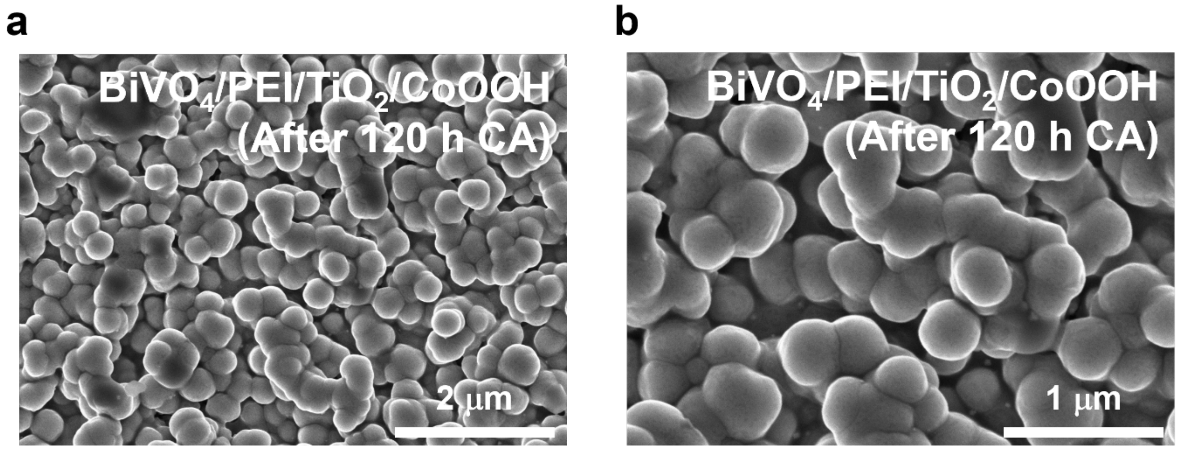

Supplementary Fig. 15. **SEM image of $BiVO_4$/PEI/TiO_2/CoOOH after the stability test. a-b,** SEM measurements revealed no significant change in the surface morphology even after 120 h stability test.

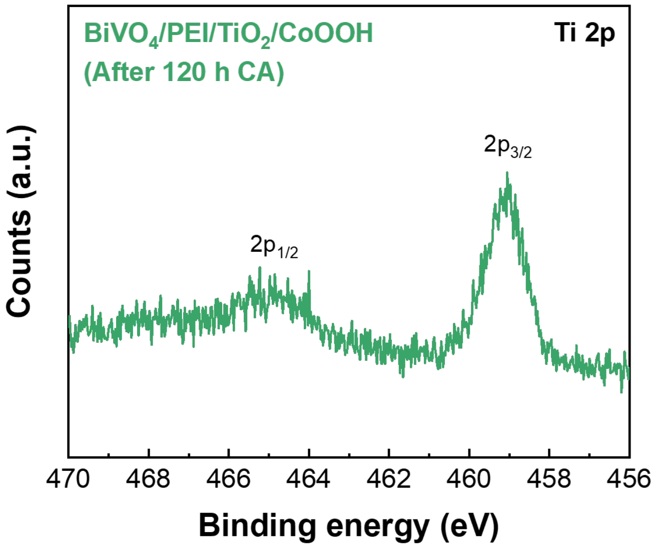

Supplementary Fig. 16. **XPS analysis of $BiVO_4$/PEI/TiO_2/CoOOH after the stability test.** High resolution XPS spectra of $BiVO_4$/PEI/TiO_2/CoOOH after 120 h stability test. XPS analysis showed clear Ti 2*p* peaks in $BiVO_4$/PEI/TiO_2/CoOOH.

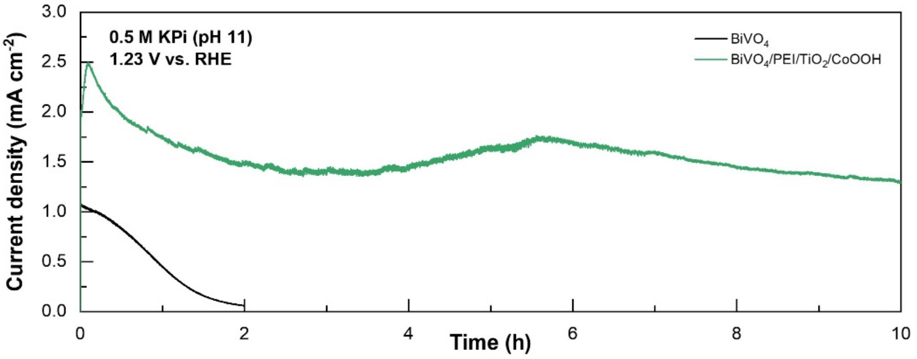

Supplementary Fig. 17. **Stability test in alkaline condition.** CA measurement of $BiVO_4$ and $BiVO_4$/PEI/TiO_2/CoOOH in alkaline electrolyte (pH 11) at 1.23 V vs. RHE.

Table S1. The comparison of photoanode stability.

Photoanode (highlighted in bold)	Electrolyte	Voltage (vs. RHE)	Photocurrent density (mA cm^{-2})	Stability (h)	Ref
**$BiVO_4$**/PEI/TiO_2 /CoOOH	0.5 M KPi (pH 8.0)	1.23 V	2.03	400	**This work**
**$BiVO_4$**/$NiFe(OH)_x$	1 M KBi (pH 9.0)	0.6 V	1.6	250	1
**$BiVO_4$**/ZCF(P)-O	1 M KBi (pH 9.0)	0.7 V	4.0	40	2
**$BiVO_4$**/$NiFeO_x$ /PAAM	1 M KBi (pH 9.0)	1.23 V	3.0	500	3
**Mo:$BiVO_4$**/NTO /$Fe_xNi_{1-x}O$	H_3PO_4/NaOH (pH 7.0)	1.23 V	5.6	16	4
**$BiVO_4$**/FeOOH /NiOOH	1 M KBi (pH 9.3)	0.6 V	2.8	60	5
**Fe_2O_3**/ZnO/CoTCPP/ FeOOH	1 M NaOH (pH 13.6)	1.23 V	3.07	20	6
**Fe_2O_3**/Fe_2TiO_5/LDH	1 M NaOH (pH 13.6)	1.23 V	3.54	20	7
**Ti:Fe_2O_3**/CoO_x/Ni	1 M NaOH (pH 13.6)	1.23 V	1.05	10	8
**n-Si**/SiO_x /CoO_x-$Mo_{2\%}$	1 M KBi (pH 9.5)	1.47 V	18.0	43	9
**n-Si**/ZrO_2/Ni/NiO /Ir SAs	1 M NaOH (pH 13.6)	1.23 V	27.7	130	10
**n-Si**/a-TiO_2/Ni	1 M KOH (pH 14.0)	1.8 V	30.0	600	11
**Si**/GaAs NW /a-TiO_2/NiO_x	1 M KOH (pH 14.0)	1.5 V	10.0	600	12
TiN/**Ta_3N_5** /CPF-TTB/$NiFeO_x$	1 M KOH (pH 14.0)	1.23 V	9.12	1.25	13
Al_2O_3/GaN/**Ta_3N_5** /$NiFeO_x$	1 M KOH (pH 13.8)	1.23 V	7.37	1.5	14
In:GaN/**Ta_3N_5** /Mg:GaN/NiCoFe-B_i	1 M KOH (pH 13.6)	1.0 V	9.3	2.7	15
**Mg:Ta_3N_5**/NiCoFe-B_i	1 M KOH (pH 13.6)	1.0 V	8.0	5	16

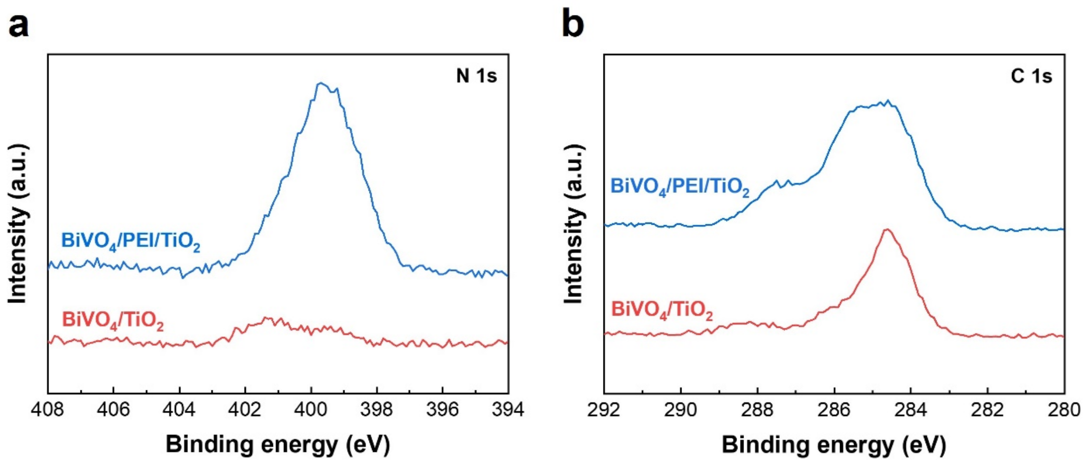

Supplementary Fig. 18. **XPS for surface characterization.** XPS analysis of N 1*s* (**a**) and C 1*s* (**b**) spectra. The increased N peak and the additional C peak were observed in $BiVO_4/PEI/TiO_2$ photoanode.

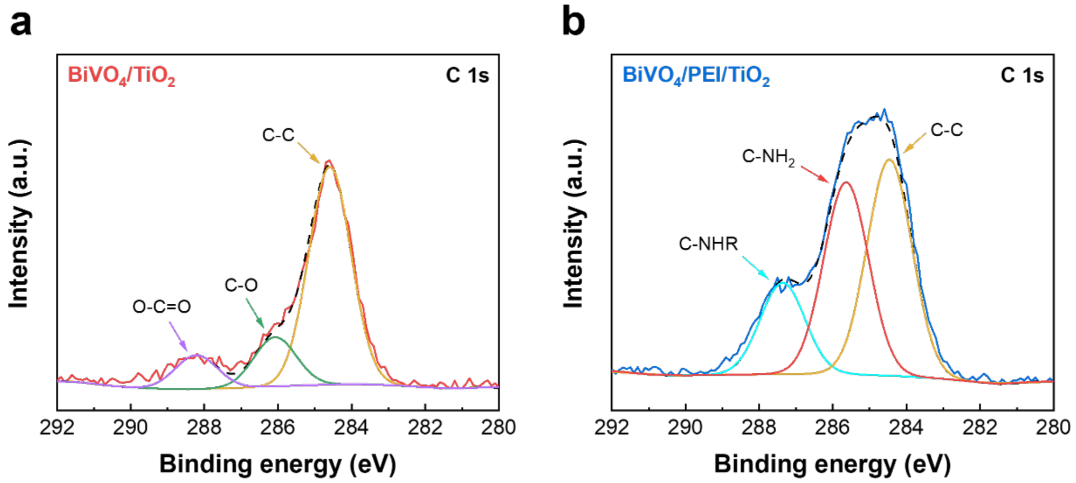

Supplementary Fig. 19. **Peak deconvolution of C 1*s* spectra in $BiVO_4/TiO_2$ and $BiVO_4/PEI/TiO_2$. a-b,** $BiVO_4/PEI/TiO_2$ showed negatively shifted peaks of C-O and O-C=O from 286.1 eV and 288.2 eV to 285.6 eV and 287.4 eV. This result indicates the presence of carbon bonded to amine-based functional groups. Specifically, the shifted peaks at 285.6 eV and 287.4 eV correspond to C-NH_2 and C-NHR functional groups, respectively.

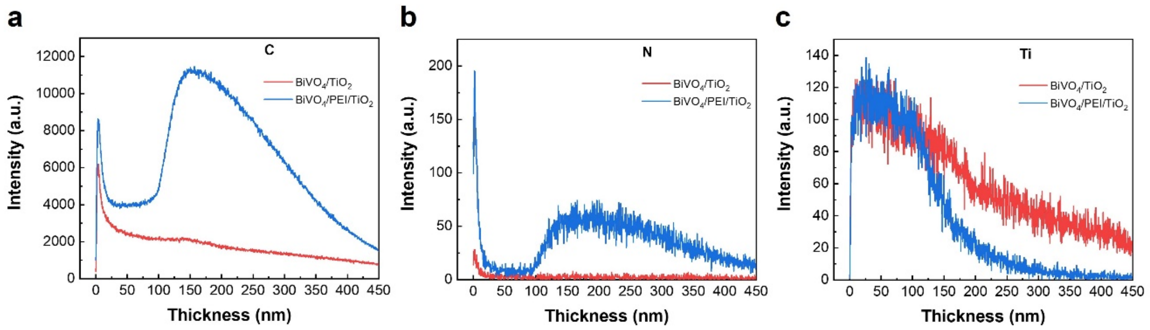

Supplementary Fig. 20. **Depth profiling analysis of $BiVO_4/TiO_2$ and $BiVO_4/PEI/TiO_2$.** TOF-SIMS analysis of C (**a**), N (**b**), and Ti (**c**) of the corresponding photoanodes.

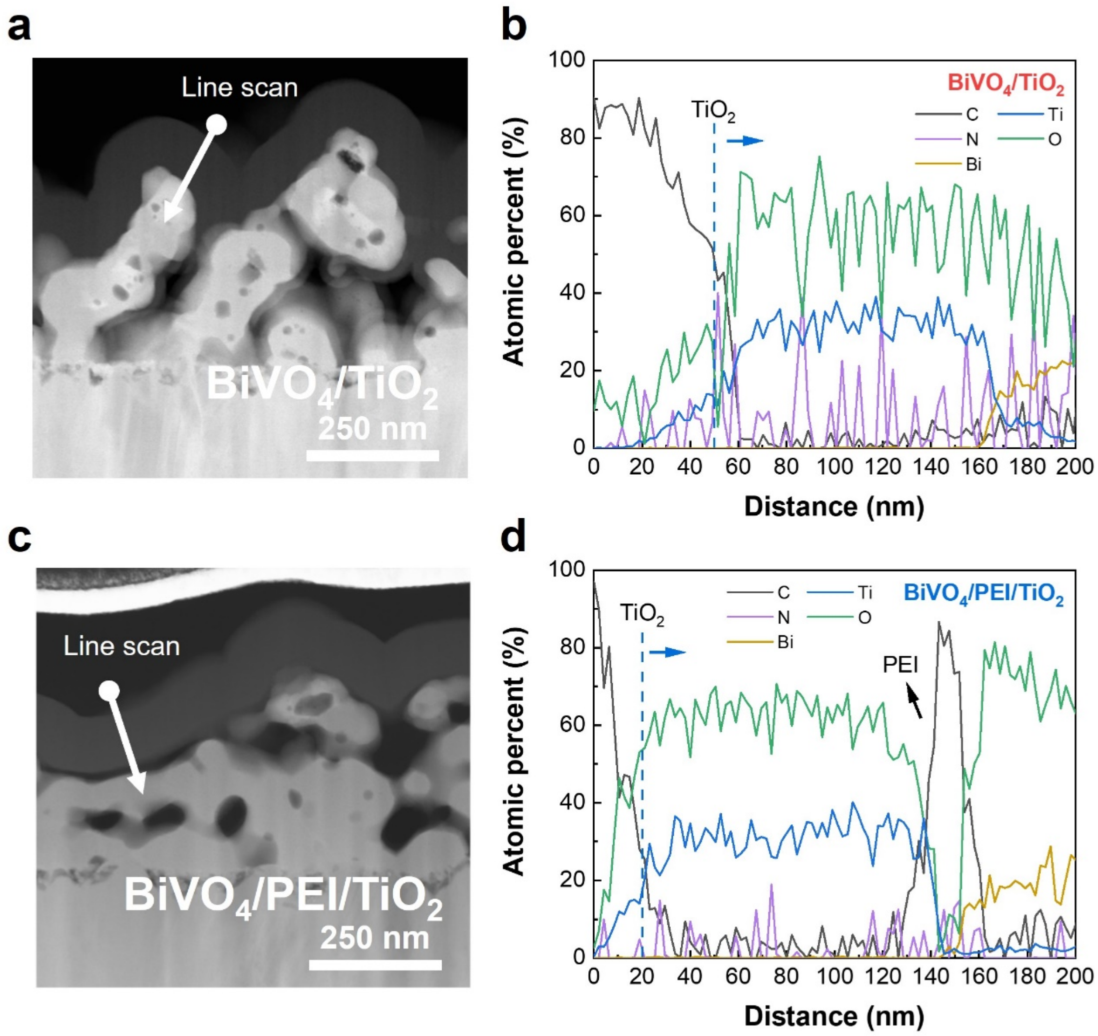

Supplementary Fig. 21. **EDX analysis for confirming configuration of photoanodes.** Line scan EDX analysis of $BiVO_4/TiO_2$ (**a,b**) and $BiVO_4$/PEI/TiO_2 (**c,d**). The presence of carbon at the interface between $BiVO_4$ and TiO_2 was exclusively observed in the $BiVO_4$/PEI/TiO_2 photoanode.

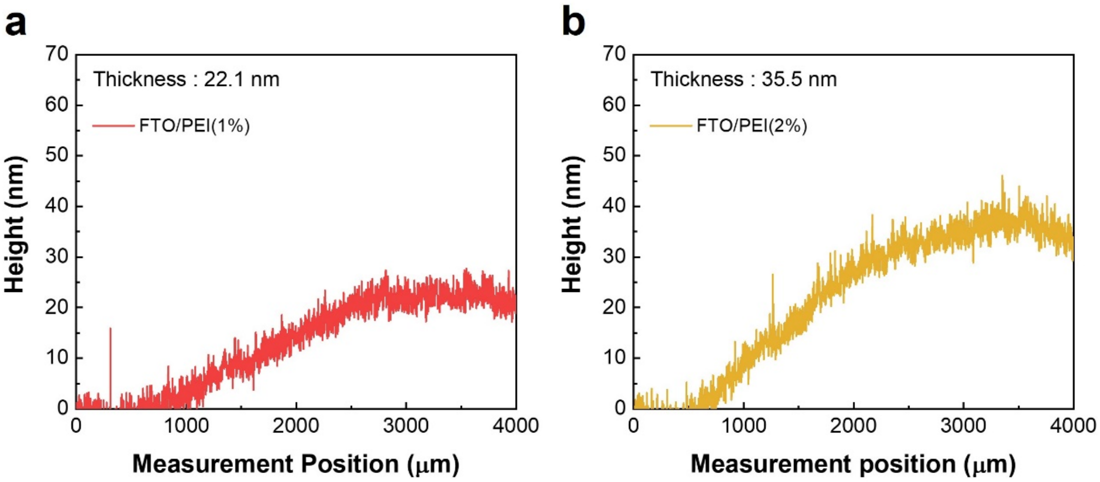

Supplementary Fig. 22. **Surface profile measurement.** Surface profilometry for measuring a thickness of PEI on FTO substrate. The thickness of the PEI layer was controlled by spin-coating using 1 wt.% (**a**) and 2 wt.% (**b**) concentrations of PEI solution.

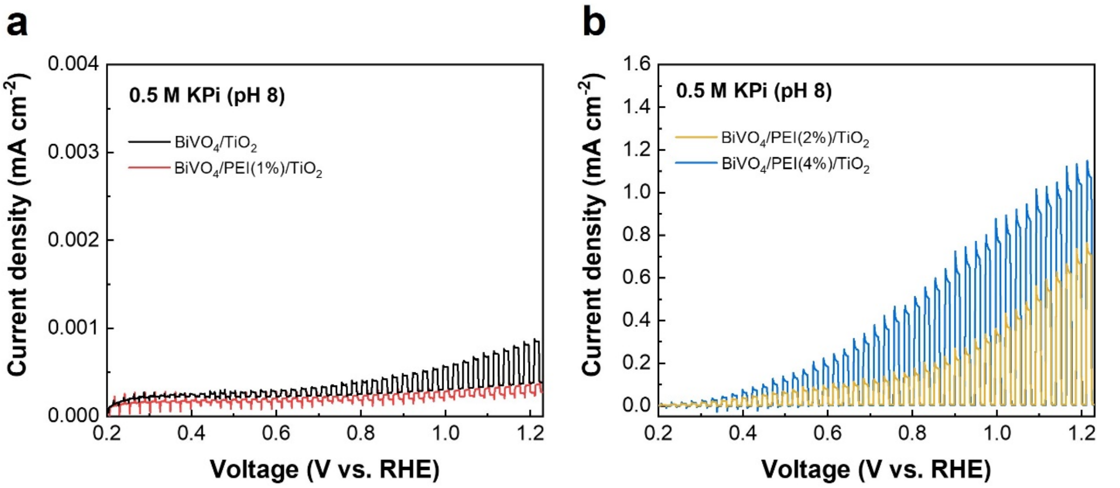

Supplementary Fig. 23. **LSV for evaluating PEC performance of photoanodes depending on the PEI thickness.** PEC water oxidation of $BiVO_4/TiO_2$ photoanodes with and without an interfacial PEI layer. The PEI layer was deposited by spin-coating with 1 wt.%, 2 wt.%, and 4 wt.% PEI solution. The photoanode modified with 1 wt.% PEI solution showed very low photocurrent (**a**), while modifications with 2 wt.% and 4 wt.% PEI solutions exhibited enhanced PEC performance (**b**).

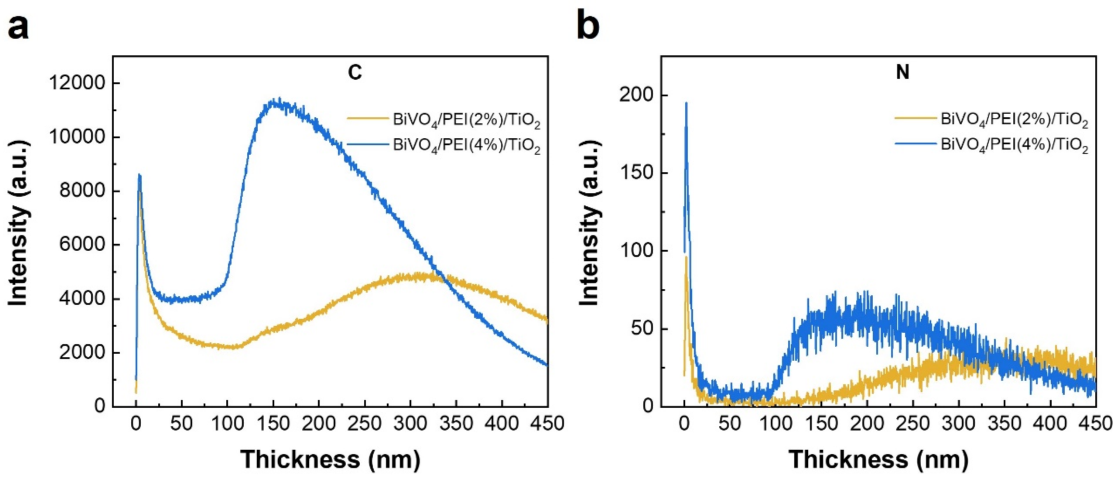

Supplementary Fig. 24. **Depth profiling analysis for confirming the amount of C and N.** TOF-SIMS analysis of $BiVO_4$/PEI(2%)/TiO_2 and $BiVO_4$/PEI(4%)/TiO_2. The intensity of C (**a**) and N (**b**) was proportional to the thickness of the PEI layer.

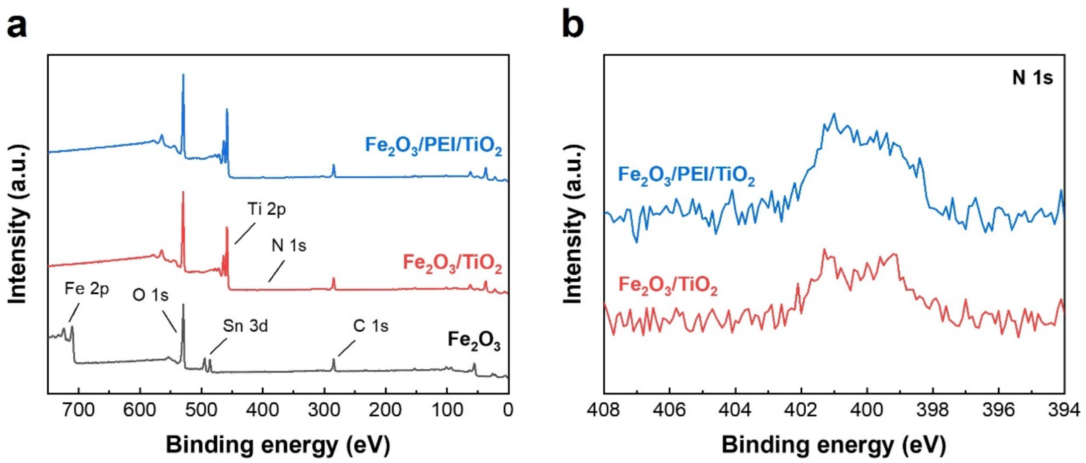

Supplementary Fig. 25. **XPS for surface characterization of Fe_2O_3 photoanodes.** XPS survey spectra (**a**) of the corresponding Fe_2O_3 photoanodes and N 1*s* peak (**b**) of Fe_2O_3/TiO_2 and Fe_2O_3/PEI/TiO_2. After deposition of 100 nm TiO_2, the Fe (2*p*) peak disappeared, and the increased N peak was observed in Fe_2O_3/PEI/TiO_2.

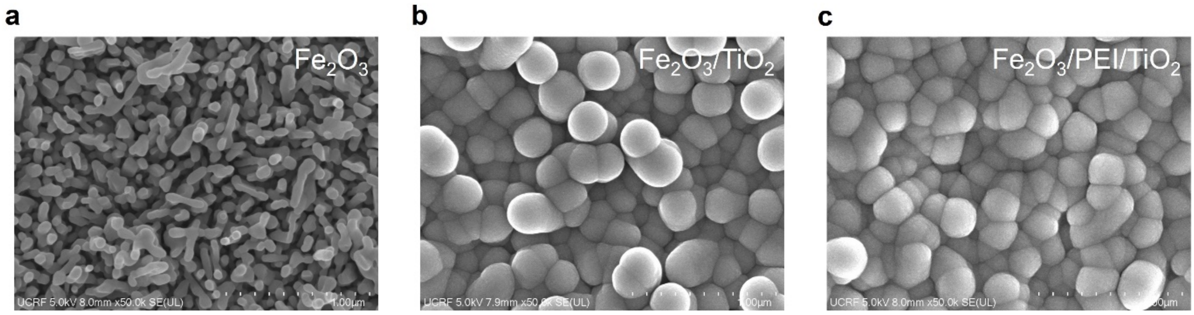

Supplementary Fig. 26. **Morphological analysis of Fe_2O_3 photoanodes.** SEM image of Fe_2O_3 (**a**), Fe_2O_3/TiO_2 (**b**), and Fe_2O_3/PEI/TiO_2 (**c**). SEM measurement revealed that the Fe_2O_3/PEI/TiO_2 appeared more compact surface than Fe_2O_3/TiO_2.

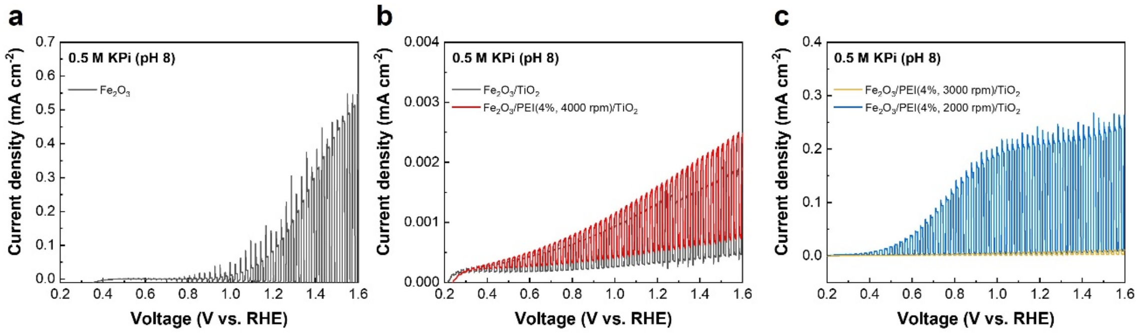

Supplementary Fig. 27. **LSV for evaluating PEC performance of Fe_2O_3 photoanodes depending on the PEI thickness.** PEC water oxidation of bare Fe_2O_3 (**a**) and Fe_2O_3/TiO_2 photoanodes with and without an interfacial PEI layer (**b,c**). The thickness of PEI layer was adjusted by varying spin-coating rate.

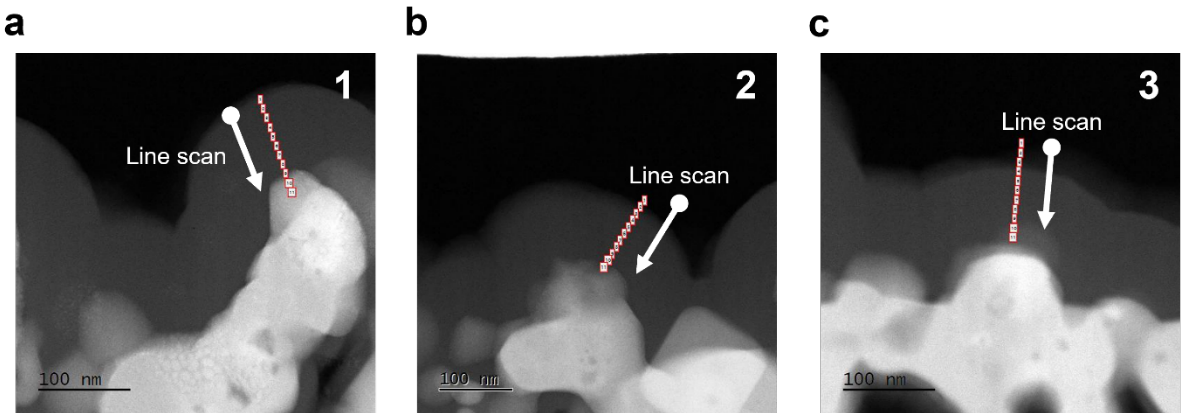

Supplementary Fig. 28. **Cross-sectional TEM images of $BiVO_4/TiO_2$ for EELS analysis. a-c,** Cs-corrected TEM measurements of $BiVO_4/TiO_2$ with the probing path of line scan for EELS.

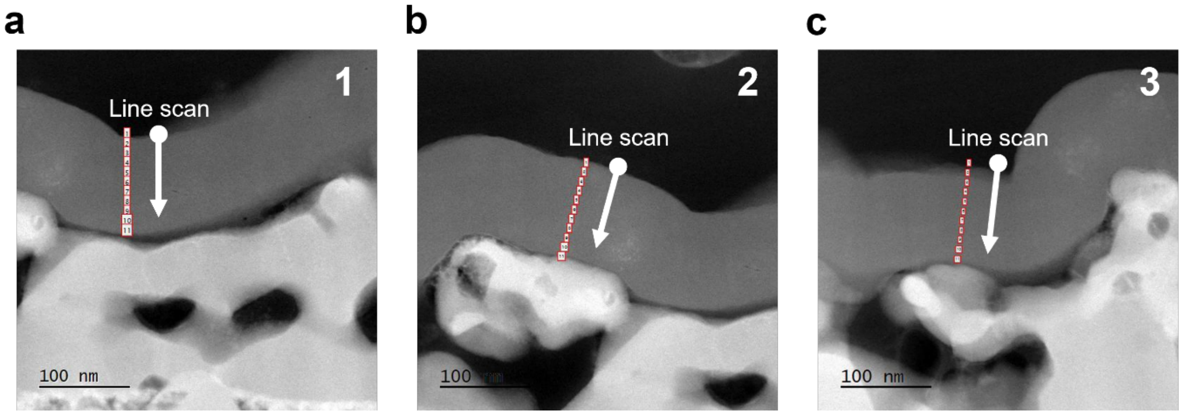

Supplementary Fig. 29. **Cross-sectional TEM images of $BiVO_4$/PEI/TiO_2 for EELS analysis. a-c,** Cs-corrected TEM measurements of $BiVO_4$/PEI/TiO_2 with the probing path of line scan for EELS.

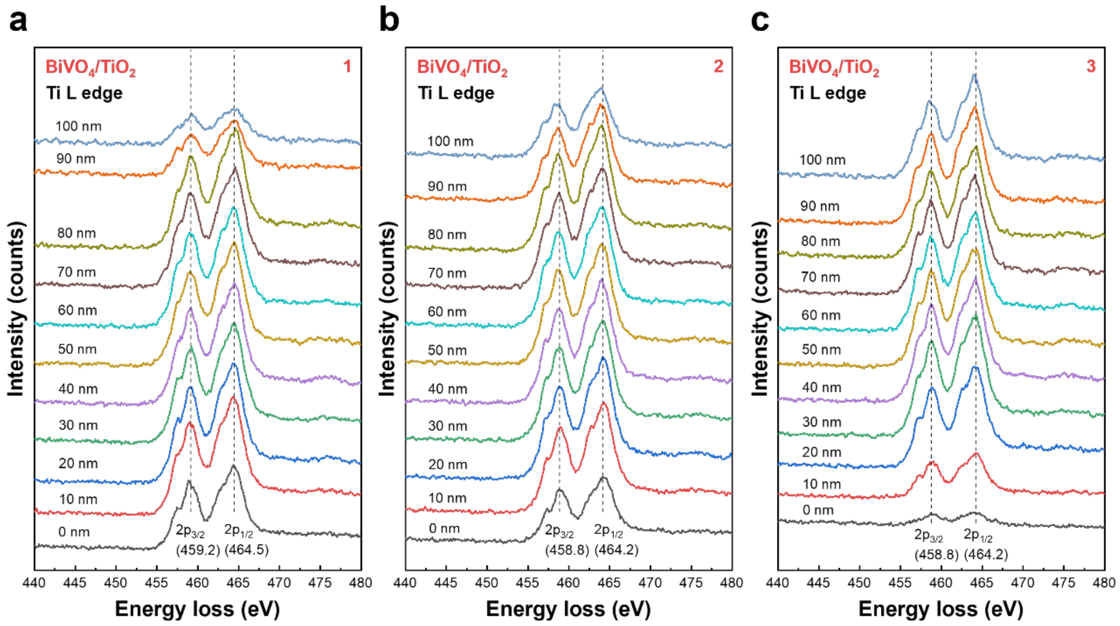

Supplementary Fig. 30. **The EELS spectra of $BiVO_4/TiO_2$. a-c,** The EELS spectra of Ti L edge obtained from line scans of three different regions in the $BiVO_4/TiO_2$ sample.

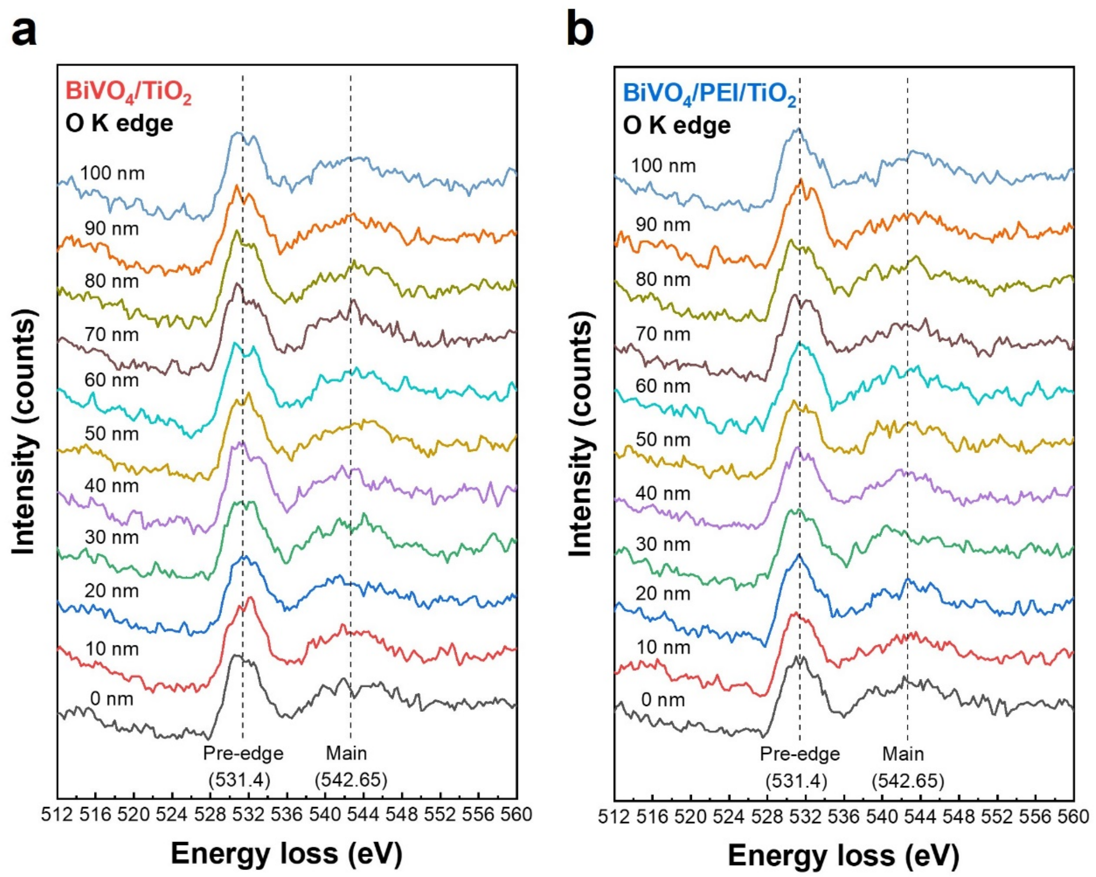

Supplementary Fig. 31. **EELS analysis for confirming oxidation state.** The EELS spectra of the O K edge obtained from the certain region of $BiVO_4/TiO_2$ (**a**) and $BiVO_4/PEI/TiO_2$ (**b**).

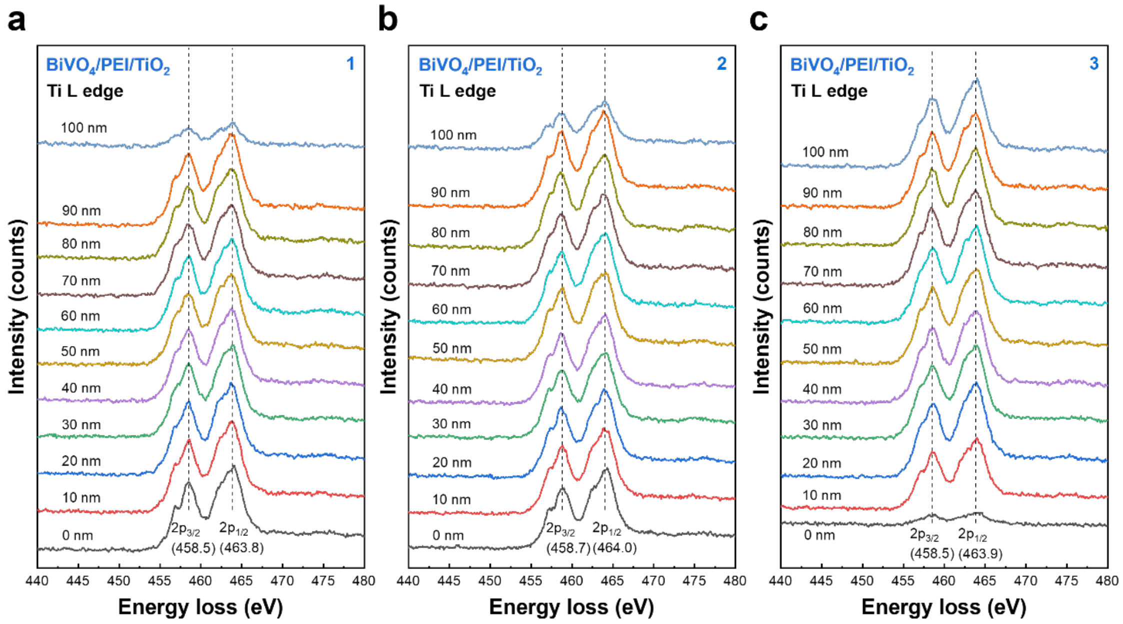

Supplementary Fig. 32. **The EELS spectra of $BiVO_4$/PEI/TiO_2. a-c,** The EELS spectra of Ti L edge obtained from line scans of three different regions in the $BiVO_4$/PEI/TiO_2 sample.

Table S2. The average position of the Ti 2*p* peak of $BiVO_4/TiO_2$ in the EELS analysis.

$BiVO_4/TiO_2$	$2p_{3/2}$	$2p_{1/2}$
1	459.2	464.5
2	458.8	464.2
3	458.8	464.2
Average	458.93	464.3

Table S3. The average position of the Ti 2*p* peak of $BiVO_4/PEI/TiO_2$ in the EELS analysis.

$BiVO_4/PEI/TiO_2$	$2p_{3/2}$	$2p_{1/2}$
1	458.5	463.8
2	458.7	464.0
3	458.5	463.9
Average	458.57	463.9

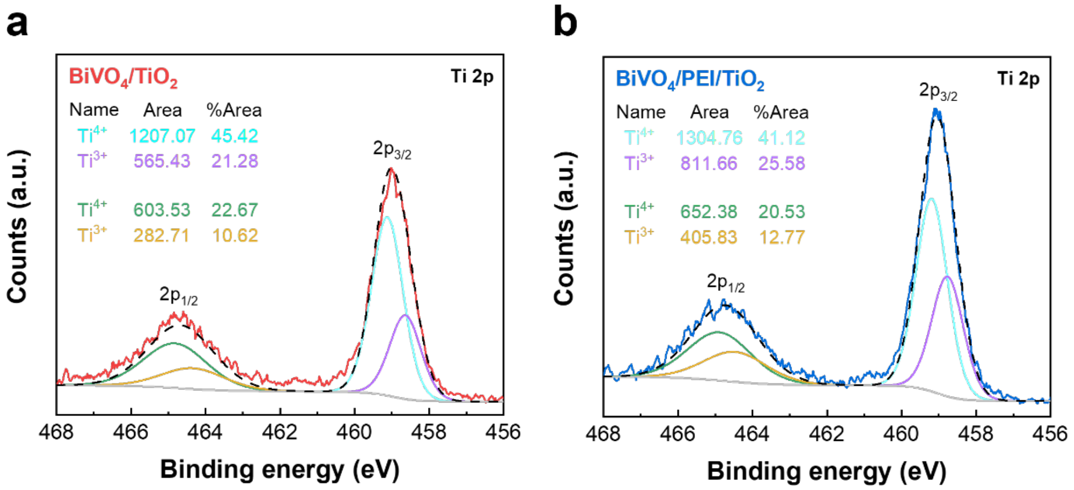

Supplementary Fig. 33. **High resolution XPS spectra of the Ti 2*p* peak in $BiVO_4/TiO_2$ and $BiVO_4/PEI/TiO_2$. a-b,** The Ti 2*p* peak deconvolution of $BiVO_4/TiO_2$ (**a**) and $BiVO_4/PEI/TiO_2$ (**b**). $BiVO_4/PEI/TiO_2$ showed a slightly higher proportion of Ti^{3+} compared to $BiVO_4/TiO_2$. To confirm the peak proportion, peak fitting was carried out under the following conditions: Smoothing of the raw data, Shirley background subtraction with an average width of 1 (dimensionless fitting parameter), an area constraint ratio of 1:2 between $2p_{1/2}$ and $2p_{3/2}$, and spin-orbit splitting of 5.7 eV for the Ti^{4+} and Ti^{3+} peaks in TiO_2.

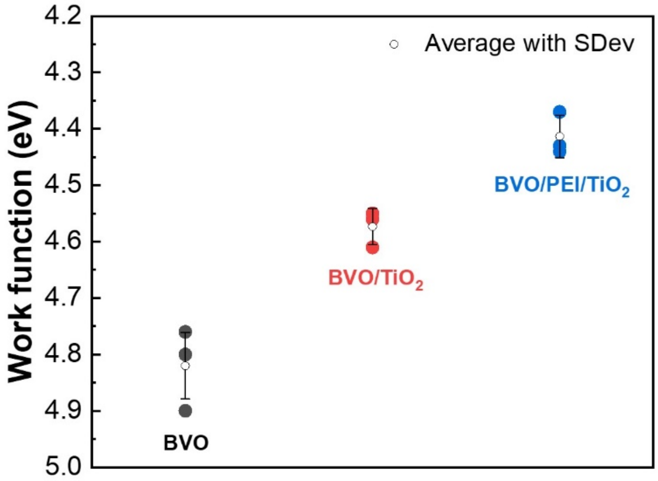

Supplementary Fig. 34. **KPFM analysis for evaluating the Fermi level of each photoanode.** Work function of each photoanode in KFPM measurement. Each work function value was determined by averaging the results from three different samples. The error bars represent the standard deviations of triplicate experiments.

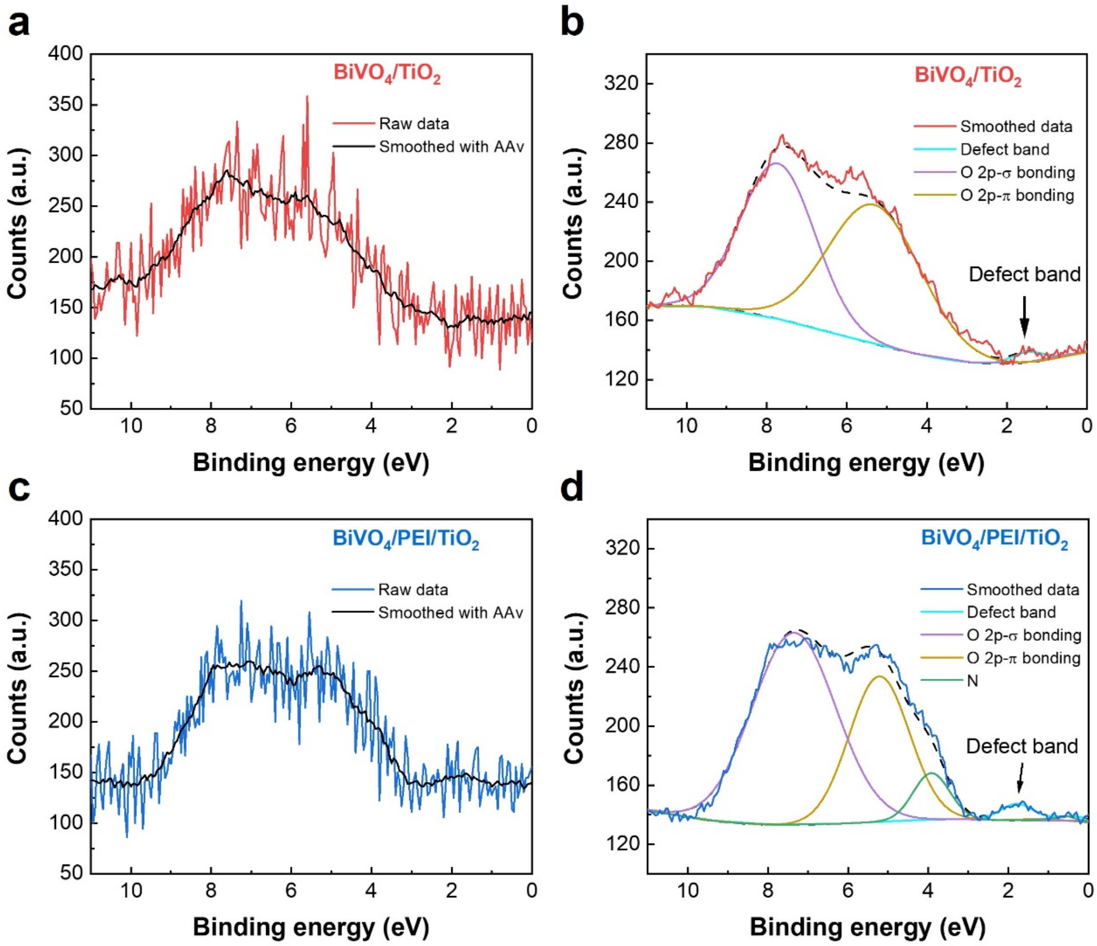

Supplementary Fig. 35. Valence state XPS analysis to elucidate the electronic structure of valence band and defect band in TiO_2. Valence state XPS measurement of $BiVO_4/TiO_2$ (**a,b**) and $BiVO_4/PEI/TiO_2$ (**c,d**). The XPS data was smoothed by adjacent averaging method (AAv). The valence state XPS analysis was measured in nearby the Fermi level region (0 eV) of the photoanodes. The additional peak in the valence band of $BiVO_4/PEI/TiO_2$ is presumed due to nitrogen from the embedded PEI.

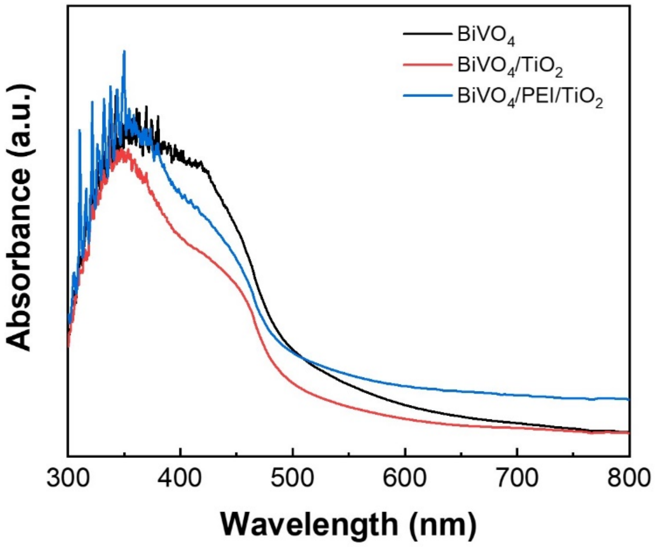

Supplementary Fig. 36. **UV-Vis spectroscopy to investigate the changed properties of the hybrid TiO_2.** Absorbance The absorbance of each photoanode was obtained by transmittance measurement. Long absorbance tail of $BiVO_4$/PEI/TiO_2 implies new Ti^{3+} states in hybrid PEI/TiO_2, originating from the embedded nitrogen into amorphous TiO_2 structure.

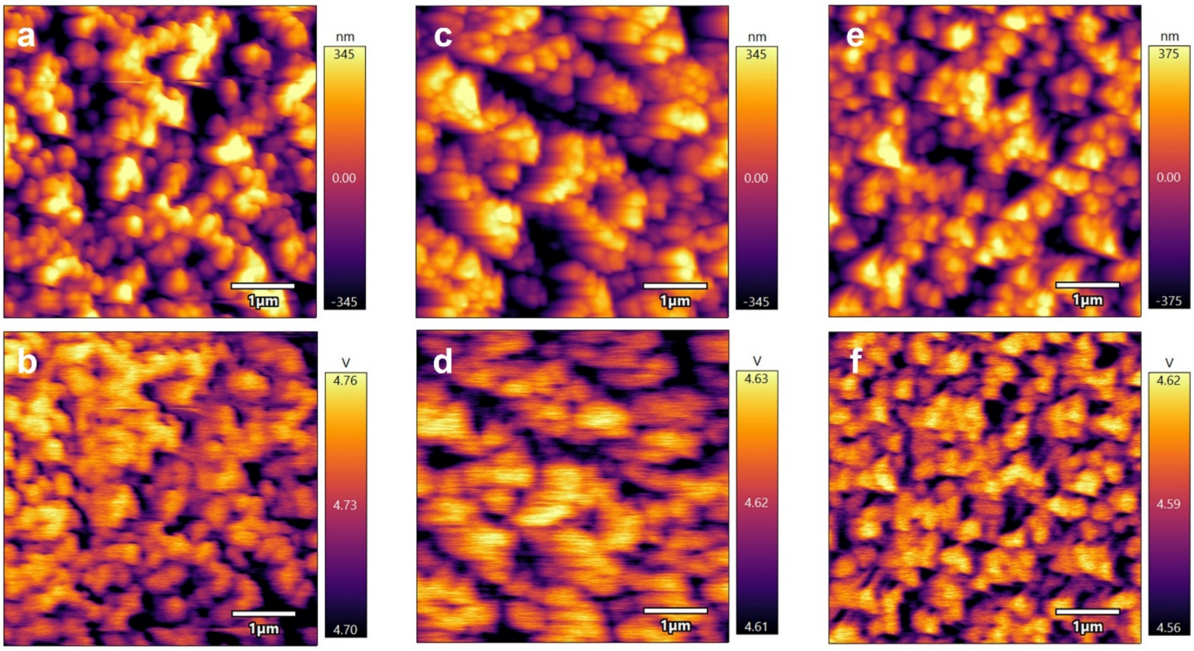

Supplementary Fig. 37. **KPFM measurement for confirming surface topology and work function.** Topography and KPFM images of $BiVO_4$ (**a,b**), $BiVO_4$/PEI(2%) (**c,d**), and $BiVO_4$/PEI(4%) (**e,f**). After deposition of PEI layer, $BiVO_4$/PEI(2%) and $BiVO_4$/PEI(4%) electrodes showed reductions in work function by 130 mV and 140 mV, respectively.

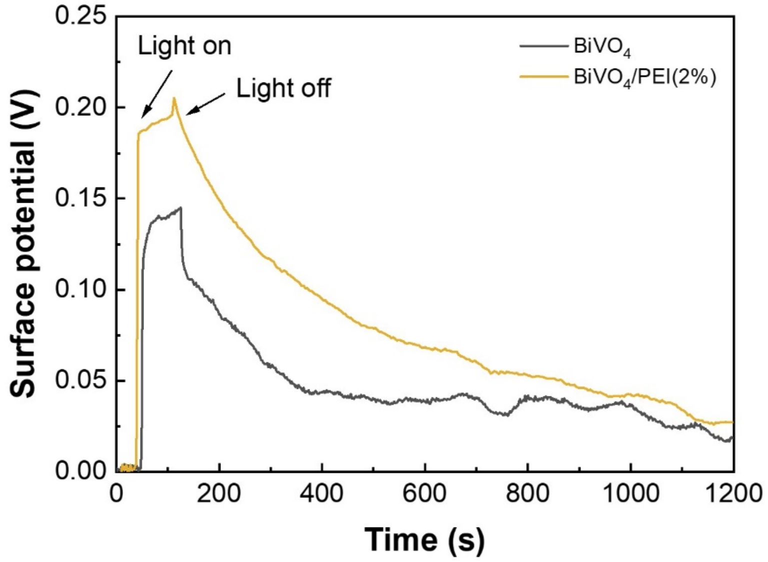

Supplementary Fig. 38. **KPFM analysis under dark and light illumination.** KPFM measurement to investigate charge-trapping of properties of PEI polyelectrolyte.

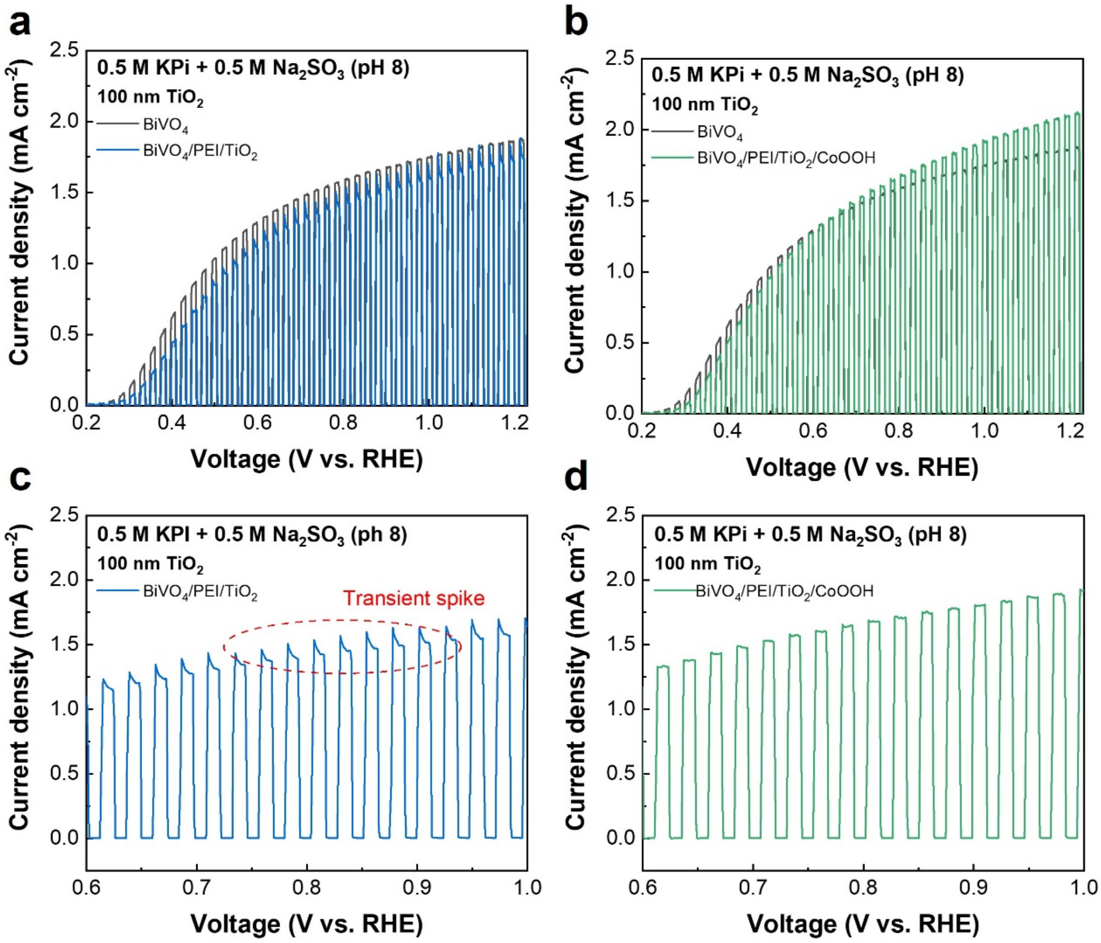

Supplementary Fig. 39. **Na_2SO_3 oxidation to investigate charge transfer kinetics. a-d,** PEC hole scavenger (Na_2SO_3) oxidation of $BiVO_4$/PEI/TiO_2 (**a,c**) and $BiVO_4$/PEI/TiO_2/CoOOH (**b,d**). The magnified LSV curves showed a transient spike in $BiVO_4$/PEI/TiO_2 photoanode without co-catalysts.

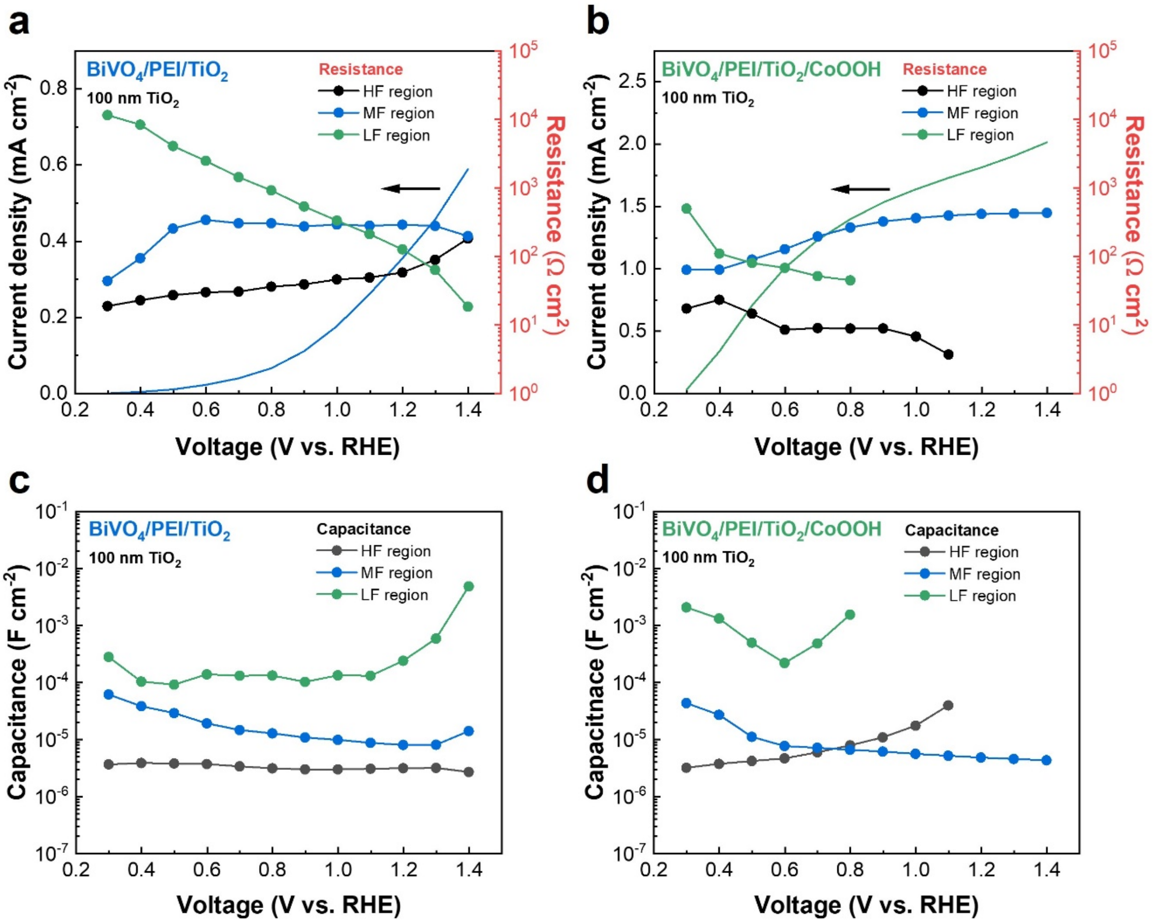

Supplementary Fig. 40. **EIS analysis to elucidate water oxidation kinetics of photoanodes with and without CoOOH.** EIS measurement measured with 0.1 V intervals under PEC water oxidation conditions (0.3~1.4 V). **a-d,** The resistance and the capacitance values of $BiVO_4$/PEI/TiO_2 (**a,c**) and $BiVO_4$/PEI/TiO_2/CoOOH (**b,d**) depending on frequency region and applied potential. High-frequency (HF), medium-frequency (MF), and low-frequency (LF) regions indicate bulk $BiVO_4$, $BiVO_4$ surface, and TiO_2 surface, respectively.

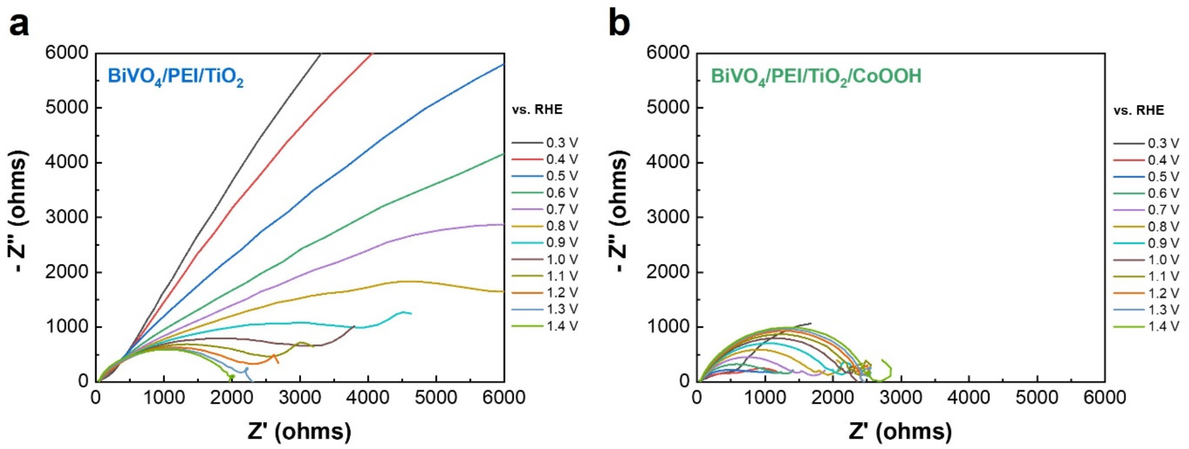

Supplementary Fig. 41. **Nyquist plots measured within the voltage range of PEC water oxidation.** Nyquist plot of $BiVO_4$/PEI/TiO_2 (**a**) and $BiVO_4$/PEI/TiO_2/CoOOH (**b**).

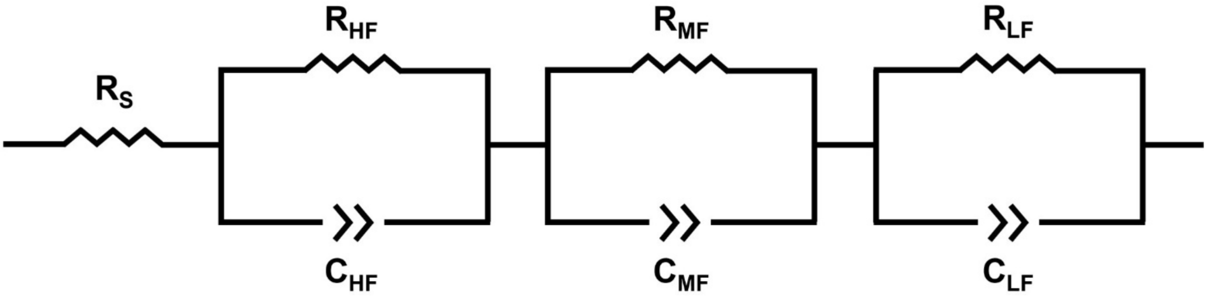

Supplementary Fig. 42. **Equivalent circuit model used for the fitting of Nyquist plots.** 3RC-equivalent circuit for fitting EIS data of each photoanode.

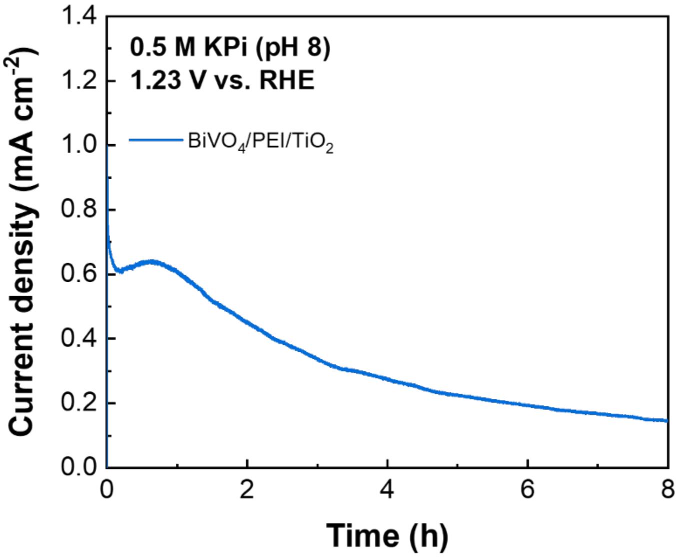

Supplementary Fig. 43. **Stability test of $BiVO_4$/PEI/TiO_2 without co-catalyst.** The stability of $BiVO_4$/PEI/TiO_2 without co-catalyst was evaluated by CA measurement at 1.23 V vs. RHE.

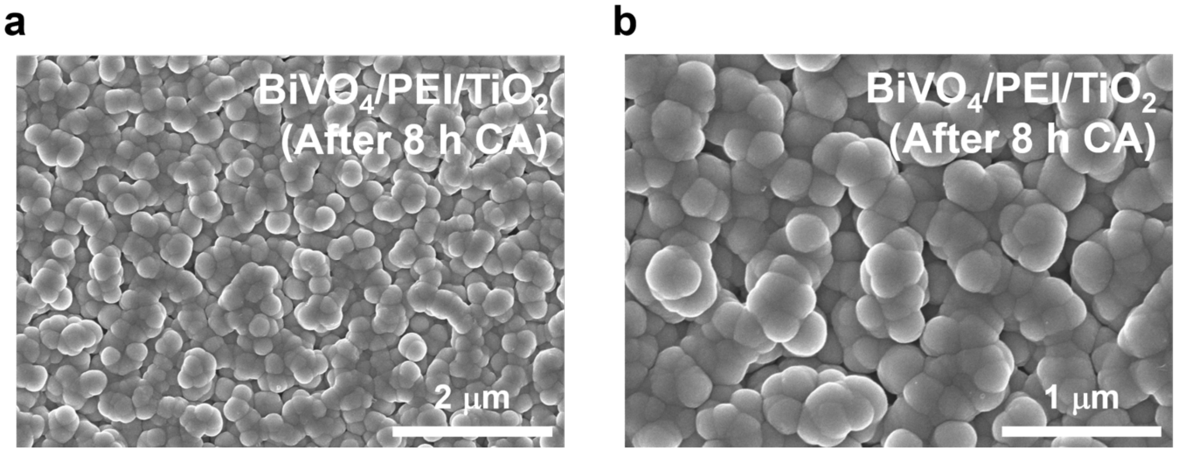

Supplementary Fig. 44. **SEM image of $BiVO_4$/PEI/TiO_2 after stability test. a-b,** SEM measurement revealed that a conformal and dense hybrid PEI/TiO_2 remained intact, even after $BiVO_4$/PEI/TiO_2 reached nearly zero current density following an 8 h stability test.

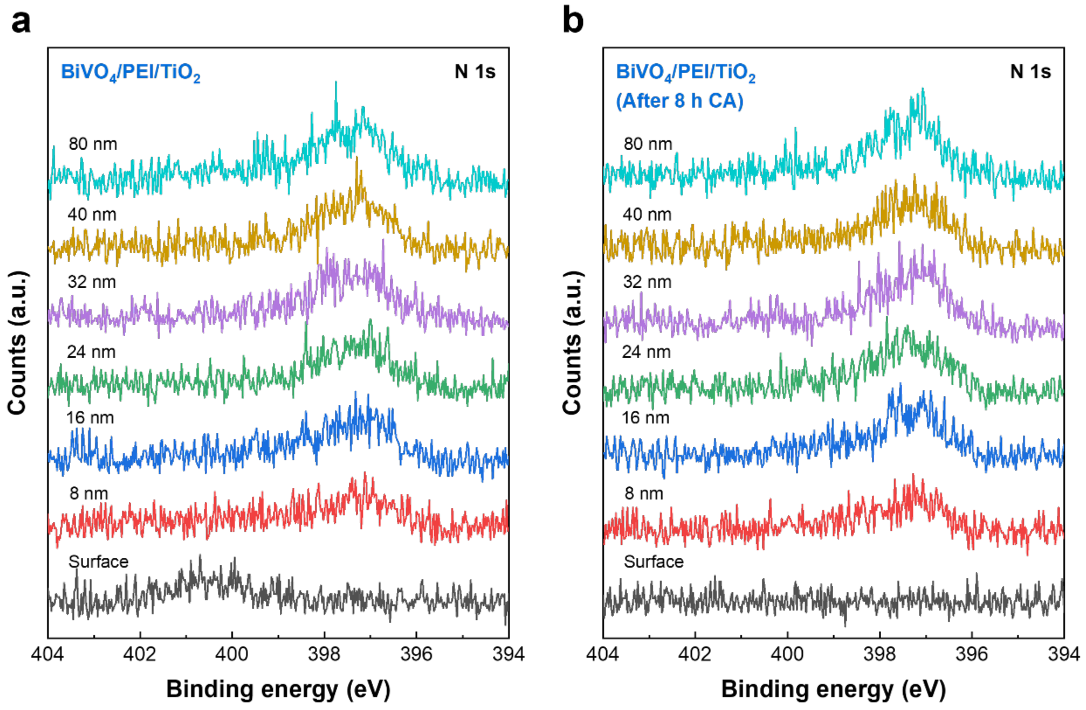

Supplementary Fig. 45. XPS depth profiling of $BiVO_4$/PEI/TiO_2 before and after the stability test. a-b, Nitrogen species are observed in both samples. We cannot be sure about the identity of the nitrogen species within the film since the depth profiling process often alters chemical states. Nevertheless, the depth profiling analysis showed identical intensities of nitrogen species in the hybrid PEI/TiO_2 before and after the stability test. This result suggests that the oxidation of the interfacial PEI layer is the main cause of decreased PEC performance of $BiVO_4$/PEI/TiO_2 during water oxidation. We assume that the interfacial PEI layer, including a small amount of internal water will be oxidized during the water oxidation reaction.

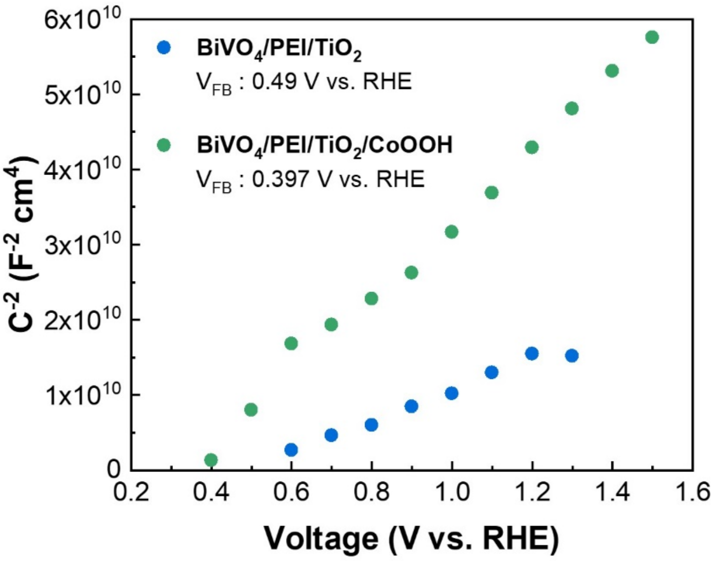

Supplementary Fig. 46. **Mott-Schottky plot obtained from the medium-frequency region of the EIS analysis.** Mott-Schottky plot of $BiVO_4$/PEI/TiO_2 and $BiVO_4$/PEI/TiO_2/CoOOH under illumination. Each photoanode showed a flat band potential near its onset potential region, which indicates the interfacial PEI layer could be electrically connected under light conditions.

Supplementary References

1 Wang, S. *et al.* Decoupled crystallization and particle growth of $BiVO_4$ via rapid thermal process for enhanced charge separation. *Adv. Funct. Mater.* 2403019 (2024).

2 Pan, J. B. *et al.* Introducing bidirectional axial coordination into $BiVO_4$@metal phthalocyanine core-shell photoanodes for efficient water oxidation. *Angew. Chem. Int. Ed.* **62**, e202307246 (2023).

3 Tan, J. *et al.* Hydrogel protection strategy to stabilize water-splitting photoelectrodes. *Nat. Energy* **7**, 537-547 (2022).

4 Beetz, M. *et al.* Ultra-thin protective coatings for sustained photoelectrochemical water oxidation with Mo:$BiVO_4$. *Adv. Funct. Mater.* **31**, 2011210 (2021).

5 Lee, D. K. *et al.* Enhancing long-term photostability of $BiVO_4$ photoanodes for solar water splitting by tuning electrolyte composition. *Nat. Energy* **3**, 53-60 (2017).

6 Xie, H. *et al.* Engineering surface passivation and hole transport layer on hematite photoanodes enabling robust photoelectrocatalytic water oxidation. *ACS Nano* **18**, 5712-5722 (2024).

7 Fouemina, J. C. N. *et al.* Surface self-transforming FeTi-LDH overlayer in Fe_2O_3/Fe_2TiO_5 photoanode for improved water oxidation. *Small* **19**, e2301114 (2023).

8 Mao, L. *et al.* Synergy of ultrathin CoO_x overlayer and nickel single atoms on hematite nanorods for efficient photo-electrochemical water splitting. *Small* **19**, e2203838 (2023).

9 Peng, S. *et al.* n-Si/SiO_x/CoO_x-Mo photoanode for efficient photoelectrochemical water oxidation. *Small* **20**, e2304376 (2024).

10 Jun, S. E. *et al.* Atomically dispersed iridium catalysts on silicon photoanode for efficient photoelectrochemical water splitting. *Nat. Commun.* **14**, 609 (2023).

11 Dong, Y. *et al.* Substantial lifetime enhancement for Si-based photoanodes enabled by amorphous TiO_2 coating with improved stoichiometry. *Nat. Commun.* **14**, 1865 (2023).

12 Shen, X. *et al.* Defect-tolerant TiO_2-coated and discretized photoanodes for >600 h of stable photoelectrochemical water oxidation. *ACS Energy Lett.* **6**, 193-200 (2020).

13 Yang, J. W. *et al.* Conjugated polythiophene frameworks as a hole-selective layer on Ta_3N_5 photoanode for high-performance solar water oxidation. *Adv. Funct. Mater.* 2400806 (2024).

14 Higashi, T. *et al.* Design of semitransparent tantalum nitride photoanode for efficient and durable solar water splitting. *Energy Environ. Sci.* **15**, 4761-4775 (2022).

15 Fu, J. *et al.* Interface engineering of Ta_3N_5 thin film photoanode for highly efficient photoelectrochemical water splitting. *Nat. Commun.* **13**, 729 (2022).

16 Xiao, Y. *et al.* Band structure engineering and defect control of Ta_3N_5 for efficient photoelectrochemical water oxidation. *Nat. Catalysis* **3**, 932-940 (2020).
